# RACIAL DISPARITIES IN OCCUPATIONAL EXPOSURES AND LATER-LIFE HEALTH

**DOI:** 10.1093/geroni/igad104.0859

**Published:** 2023-12-21

**Authors:** Amanda Sonnega, Theresa Andrasfay

## Abstract

Racial and ethnic health disparities remain a persistent and urgent social problem. Researchers are beginning to explore the role of work experiences, both good and bad, in attenuating or exacerbating health disparities. The recent availability of the entire life course experience of work in the Health and Retirement Study (HRS) Life History Mail Survey (LHMS) has spurred innovative research on the role of occupational exposures on health disparities in later life. Linking these data to the information available in the Occupation Information Network (O*NET) provides a rich characterization of lifetime occupational exposures. Sheftel et al. use work histories from the LHMS to study the role of racial segregation in occupations on later life dementia. They show that complex work with data during working ages may be protective against dementia at older ages, potentially contributing to the differentials in dementia prevalence by race/ethnicity. Park et al. similarly use data on lifetime work histories, to investigate the role of accumulated years of physically demanding work (PDW) in young and middle adulthood on the number of functional limitations at age 60. They show that cumulative PDW is strongly associated with functional limitations at age 60 and accounts for a part of the racial/ethnic gap in functional limitations at age 60. Both papers will discuss policy implications of findings. Andrasfay will provide critical commentary and discussion.

